# Status and determinants of intra-household food allocation in rural Nepal

**DOI:** 10.1038/s41430-017-0063-0

**Published:** 2018-01-22

**Authors:** Helen A. Harris-Fry, Puskar Paudel, Niva Shrestha, Tom Harrisson, B. James Beard, Sonali Jha, Bhim P. Shrestha, Dharma S. Manandhar, Anthony M. D. L. Costello, Mario Cortina-Borja, Naomi M. Saville

**Affiliations:** 1 London School of Hygiene and Tropical Medicine, London, UK; 2 Institute for Global Health, University College London, London, UK; 3 Mother and Infant Research Activities, Kathmandu, Nepal; 4 Maternal Child and Adolescent Health, World Health Organization, Geneva, Switzerland; 5 Great Ormond Street Institute of Child Health, University College London, London, UK

**Keywords:** Malnutrition, Epidemiology

## Abstract

**Background/objectives:**

Understanding of the patterns and predictors of intra-household food allocation could enable nutrition programmes to better target nutritionally vulnerable individuals. This study aims to characterise the status and determinants of intra-household food and nutrient allocation in Nepal.

**Subjects/methods:**

Pregnant women, their mothers-in-law and male household heads from Dhanusha and Mahottari districts in Nepal responded to 24-h dietary recalls, thrice repeated on non-consecutive days (*n* = 150 households; 1278 individual recalls). Intra-household inequity was measured using ratios between household members in food intakes (food shares); food-energy intake proportions (‘food shares-to-energy shares’, FS:ES); calorie-requirement proportions (‘relative dietary energy adequacy ratios’, RDEARs) and mean probability of adequacy for 11 micronutrients (MPA ratios). Hypothesised determinants were collected during the recalls, and their associations with the outcomes were tested using multivariable mixed-effects linear regression models.

**Results:**

Women’s diets (pregnant women and mothers-in-law) consisted of larger FS:ES of starchy foods, pulses, fruits and vegetables than male household heads, whereas men had larger FS:ES of animal-source foods. Pregnant women had the lowest MPA (37%) followed by their mothers-in-law (52%), and male household heads (57%). RDEARs between pregnant women and household heads were 31% higher (log-RDEAR coeff=0.27 (95% CI 0.12, 0.42), *P* < 0.001) when pregnant women earned more or the same as their spouse, and log-MPA ratios between pregnant women and mothers-in-law were positively associated with household-level calorie intakes (coeff=0.43 (0.23, 0.63), *P* < 0.001, per 1000 kcal).

**Conclusions:**

Pregnant women receive inequitably lower shares of food and nutrients, but this could be improved by increasing pregnant women’s cash earnings and household food security.

## Background

Pregnant women in South Asia have inadequate intakes of many micronutrients [1, 2], and this can translate into comorbidities of multiple micronutrient deficiencies [3]. Inadequate diets during pregnancy are particularly problematic because inadequate weight gain and micronutrient intakes are associated with higher risk of adverse health outcomes, including low birth weight [4] and maternal mortality [5]. In 2013, over half of the world’s maternal deaths caused by severe anaemia occurred in South Asia [5].

In South Asia, nutritional inadequacy may be caused by gender-based inequities. At the macro level, the Gender Inequality Index displaces gross domestic product as a predictor of low birthweight, suggesting that inequality is a more important determinant of nutrition than poverty [6]. At the micro level, women [7, 8], particularly pregnant women [9], are discriminated against the allocation of food within households—a trend that is more prominent in South Asia than elsewhere [9]. This may be explained by food insecurity [10] or sociocultural factors [7]. For example, women often eat last and least [11], fast more than men [12] and have limited decision-making power over food-purchasing decisions [13]. Additionally, during pregnancy, women have higher nutritional requirements but often have other pregnancy-specific food restrictions [7].

To improve nutrition during pregnancy, many interventions have aimed to increase household-level food availability, by providing supplements, social transfers [14] or promoting home food production through gardening or livestock programmes [15]. However, if pregnant women are discriminated against, interventions may fail to benefit them.

Recent, high-quality studies on intra-household food allocation are limited [9], and none of them have used probability methods to estimate nutritional adequacy or examined inequities between pregnant women and mothers-in-law [16]. The present study from Nepal will describe intra-household allocation of food-related behaviours, food groups and dietary adequacy between pregnant women, mothers-in-law and male household heads, and use a recent theoretical framework [16] to identify determinants of intra-household food allocation.

## Subjects and methods

### Study population

The study was conducted in Dhanusha and Mahottari districts, located in Province 2, in the *Terai* (lowland) region of Nepal. Dhanusha and Mahottari districts have a combined population of ~1.4 million, and the main source of livelihood is agricultural production [17]. Located in the Indo-Gangetic floodplains, land is fertile and there are favourable climatic conditions for agricultural production; yet, the prevalence of undernutrition is the highest in the country; 29% of women in Province 2 are underweight (<18.5 kg/m^2^), compared with the national average of 17% [18].

The pre-specified sampling frame included all male-headed households, with a pregnant woman in their third trimester who was living with their mother-in-law and enroled in a cluster-randomised controlled trial: the Low Birth Weight South Asia Trial (LBWSAT; http://www.controlled-trials.com/ISRCTN75964374) [19, 20] between June and September 2015. We sampled joint, male-headed households to reduce heterogeneity and because qualitative research indicated that they would be least likely to change food allocation behaviours [13]. Within households, respondents were pregnant women, their mothers-in-law and the male household heads. Dietary data were collected from 805 households in all trial arms, based on a target sample size of 200 households from 19 clusters per arm, to detect a difference of 0.1 ‘Relative Dietary Energy Adequacy Ratios’ (RDEARs) between two trial arms with 80% power and 95% confidence. This study uses data from the control arm (*n* = 150) in 20 Village Development Committee areas.

Informed consent was obtained from all respondents and research ethics approval was obtained from the Nepal Health Research Council (108/2012) and University College London Ethical Review Committee (4198/001).

### Data collection

Interviewers collected 24-h dietary recalls using a smartphone tool, described elsewhere [21]. In brief, interviewers conducted dietary recalls, repeated three times per person on non-consecutive days, following five passes each time: collect a free recall using non-specific probes, ask the time and place that each item was consumed, read a list of commonly forgotten foods, recap in chronological order, and collect details on specific food types and portion sizes [22, 23].

Food types were selected from a precoded list of foods, including locally available supplements, or typed manually if missing from the list. Portion sizes were estimated using a photographic atlas that was validated for this study and contained 224 graduated discrete, life-sized portion images for 72 foods. We used the same images for similar foods [24]. Data were collected on Android smartphones using CommCare (Version 2.22.0, http://www.commcarehq.org/home/), an open-source, cloud-based data collection platform. Codes for food items and portions were encoded in quick response (QR) codes and entered into the form using a barcode scanning application (‘ZXing Barcode Scanner’). To minimise non-response, pregnant women could respond on behalf of others if they felt confident answering comprehensively. This was not permitted during the first visit when anthropometric measurements were taken. The nutritional composition of raw foods was calculated using a Food Composition Table (FCT) compiled from multiple sources [25–28]. For mixed dishes, we calculated the average nutritional composition from 174 recipes collected prior to dietary data collection.

Body weight and mid-upper arm circumference (MUAC) were measured using Tanita solar-weighing scales and Seca 212 circumference tapes, respectively. Self-reported activity levels, illness, feasting and fasting, food security (months of adequate household food provisioning, MAHFP [29] and household food insecurity access scale, HFIAS [30]) and other diet-related questions were collected, plus the following hypothesised determinants: pregnant women earning the same or more cash than their spouses; gravidity (a proxy for seniority); self-reported empowerment level of pregnant woman (scale 0–10); asset score calculated using principal component analysis; household calorie consumption (averaged of the three members, per 1000 kcal); pregnant woman’s husband living overseas; caste or religious group; and season (pre-monsoon or during monsoon). We used other socioeconomic data collected by the main trial surveillance questionnaires [20].

### Data analysis

Foods were aggregated into the ten food groups in the minimum dietary diversity score for women (MDD-W) [31]: (1) grains, white roots and tubers, (2) pulses (beans, peas and lentils), (3) nuts and seeds, (4) dairy, (5) meat, poultry and fish, (6) eggs, (7) dark green leafy vegetables, (8) other vitamin A-rich fruits and vegetables, (9) other vegetables and (10) other fruits. We calculated MDD-W by summing the groups consumed on the first recall (to use the same reference period for which the score was validated), and calculated the proportion consuming an ‘adequate’ diet (≥5 food groups) [31].

Nutritional intakes were estimated by calculating the nutrients from each portion of each food using the FCT, and summing the nutrients from each portion to give total daily intakes. We did not apply nutrient retention factors because of the lack of locally appropriate estimates. Intakes were averaged across the three recall visits.

Dietary adequacy was calculated using the USA Institute of Medicine (IOM) probability approach [32, 33]. First, to achieve normality, nutrient intakes were transformed using a Box–Cox model [34]. Then, using transformed values, we calculated ‘usual’ intakes from the best linear unbiased predictors resulting from mixed-effects models, fitted separately for each household member type. We treated clusters and individuals as random effects and strata as fixed effects. For all nutrients (except iron for non-pregnant respondents), the probability of adequacy (PA) was calculated by comparing each back-transformed usual intake to the population distribution of requirements, which are normal distributions with means (i.e. estimated average requirements, EARs) and standard deviations. We used WHO/FAO’s values for nutritional requirements of vitamin C, thiamin, riboflavin, niacin, vitamin B_6_, folate, vitamin B_12_ [35], Institute of Medicine’s values for calcium [36] and iron [37] and International Zinc Nutrition Consultative Group (IZiNCG)’s recommendations for zinc [38]. Iron requirements for non-pregnant women and men are not normally distributed, so, we calculated PAs using a table of probabilities for different intake intervals, adapted from IOM [37] to assume 5% bioavailability. The mean probability of adequacy (MPA) was the average PA of all 11 nutrients.

To measure intra-household food allocation, we calculated food shares (FS), food-share-to-energy shares (FS:ES), RDEARs and MPA ratios. FS are ratios of food group intakes (g) between pairs of individuals for households who consumed any [39]. FS:ES account for different energy intakes between individuals [39], calculated as $\left( \rm{food\,intake_a/kcal\,intake_a} \right)/\left( \rm{food\,intake_b/kcal\,intake_b} \right)$, for persons a and b. Energy allocation was calculated as the ‘Relative Dietary Energy Adequacy Ratio’, $\rm{RDEAR} = \left( \rm{intakes_a/EAR_a} \right)/\left( {intakes_b/EAR_b} \right)$ [9]. Energy EARs were calculated according to age, gender, pregnancy status, body weight (kg) and self-reported activity levels, using values by Indian Council of Medical Research [40]. The additional cost of pregnancy was taken to be 390 kcal/d [40]. MPA ratios were calculated as $\rm{MPA_a/MPA_b}.$

To test for inequity, we adjusted for deviations from normality by log-transforming the ratios and used a random effects linear regression model, treating clusters as a random effect, to test whether the intercept was significantly different from zero.

To identify determinants of food allocation, using RDEARs and MPA ratios as outcomes, we fitted multivariable mixed-effects linear regression models, including all hypothesised determinants. We tested for nonlinear effects of wealth on log-RDEAR and log-MPA ratios [16]. To assess collinearity among predictors, we calculated variance inflation factors (VIFs) [41]. We included all outliers in kcal intakes, and respondents who were fasting or feasting because the results were comparable with analyses excluding outliers, but excluded extreme outliers (<−8) in log-transformed MPA ratios to give normally distributed residuals. Significance levels were set at *P* < 0.05.

### Code availability

All analyses were conducted using Stata SE 14 (College Station, TX: StataCorp LP) and Stata code is available upon request with the corresponding author.

## Results

We sampled 75% (150/199) of eligible households. The reasons for non-response included non-consent (*n* = 5) or non-availability (*n* = 41). Some households on the sample list were not sampled because they had become ineligible before the interview, because women had given birth (*n* = 108) or were temporarily not living with their mothers-in-law (*n* = 101). The study period also covered pre-monsoon (hottest) and monsoon, mango season and Ramadan. Cluster-adjusted chi-square tests show no significant differences in age, caste, assets, land ownership, education or HFIAS between sampled and non-sampled participants (results not shown).

Respondent characteristics are summarised in Table 1. Almost a third were landless, over a third were from disadvantaged groups (Dalit or Muslim) and over half of pregnant women had not attended school. There was some food insecurity in the month preceding the interview in 30% of households, though only 9% cited any months of inadequate household food provisioning in the preceding year. Male household heads had the lowest incidences of illness and fasting, and prevalence of low MUAC (14% < 23 cm [42]) compared with pregnant women (40%) or mothers-in-law (35%). Men and mothers-in-law were involved in food shopping and decision-making, whereas most pregnant women did the cooking (78%).

**Table 1:** Household and individual socioeconomic and demographic characteristics, and food-related behaviours

Respondent characteristics	Pregnant woman		Mother-in-law		Household head	
Age, years						
Median (25th, 75th centiles)	21	(19, 24)	50	(44, 56)	39	(25, 56)
Age at marriage, years						
Mean (SD)	16.4	(1.8)	NA		NA	
Number of previous pregnancies, %						
0	32.4		NA		NA	
≥1	67.6		NA		NA	
Gestational age, weeks						
Median (25th, 75th centiles)	37	(35, 38)	NA		NA	
Mid-upper arm circumference, MUAC						
Low MUAC, <23 cm, %	40.0		35.3		14.0	
Mean (SD)	23.5	(2.1)	24.3	(3.3)	25.9	(2.9)
Illness and fasting, %						
Any illness in the three dietary recall reference periods	13.3		12.0		6.7	
Any fasting in the three dietary recall reference periods	10.0		13.3		8.7	
Ate more during pregnancy, compared to when not pregnant	15.1		NA		NA	
Ate the same during pregnancy, compared to when not pregnant	32.5		NA		NA	
Ate less during pregnancy, compared to when not pregnant	52.4		NA		NA	
Involvement in food production and preparation, %						
Main cook in the household	77.8		3.2		0	
Involved in decisions about purchasing food	16.0		50.7		50.0	
Goes outside to do the shopping	13.4		38.8		57.5	
Education level, %						
Never went to school	56.1		NA		NA	
Primary to lower secondary	27.0		NA		NA	
Secondary and above	16.9		NA		NA	
Household-level characteristics						
Caste group, %						
Dalit/Muslim (most disadvantaged groups)	36.2					
*Janajati*/other *Terai* castes	42.9					
*Yadav*/*Brahmin* (least disadvantaged)	20.9					
Land ownership, %						
Owns no land	30.9					
Household food security, %						
Households with enough food to meet household needs in the year prior to interview (MAHFP)	91.0					
Households experiencing no food insecurity over the past 4 weeks prior to interview (HFIAS)	69.4					

*n* = 150; response rates for these variables ranged from 89% (food security) to 100% (age, caste)

*NA* not available or applicable, *HFIAS* household food insecurity access scale, *MAHFP* months of adequate household food provisioning

For all household members, almost all (98%) respondents ate rice, around three quarters ate *dal* (spiced lentil soup) and 65% ate *roti* (flatbread). Other food items, that >20% of respondents consumed at least some of, were tea with sugar and milk, mango, pointed gourd curry, fried spicy potato (*bhujiya*) and buffalo milk. Only 9% of pregnant women and 32% of mothers-in-law consumed food outside of the home over the 3-day recall, compared with 73% of male household heads. Household heads commonly ate outdoors or in a teashop, and ate plain, puffed or beaten rice (18%), vegetable curry (13%), tea with sugar and milk (9%), flatbreads (9%), deep-fried sweet or savoury snacks like *samosa, litti* and *jeri* (9%), *dal* (6%) and alcohol (6%). All household members consumed around two-thirds of their calories before 11 a.m. or after 7 p.m.

### Intra-household differences in food consumption and nutrient adequacy

**Fig. 1:**
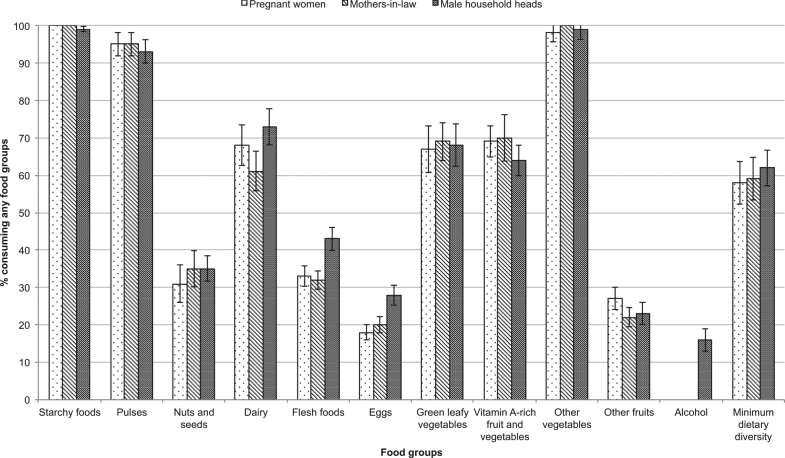
Percentage of pregnant women, mothers-in-law and male household heads consuming any of each food group, based on 3 days of dietary recall, and consuming minimum dietary diversity based on 1 day of dietary recall

The percentage of pregnant women, mothers-in-law and male household heads consuming any of the 10 food groups or alcohol, and the percentage consuming an adequate diet (≥5 food groups), is given in Fig. 1. Error bars show standard errors of the mean, adjusted for clustering. Mean intakes of those who consumed any of each group are given in Table 2. More household heads consumed animal-source foods (flesh foods like meat or fish, eggs and dairy) than pregnant women or mothers-in-law. A total of 43% of household heads consumed flesh foods compared with a third of pregnant women or mothers-in-law; 73% of household heads consumed dairy compared with 61% of mothers-in-law. More pregnant women ate green leafy vegetables or fruits than mothers-in-law or household heads. Consumption of most other foods—especially common foods like starchy foods, pulses and vegetables—and mean dietary diversity score (between 4.6 and 4.9) was similar for all three household members.

**Table 2:** Mean consumption of food groups for household members who consumed any, and mean dietary diversity score, for each household member

	Pregnant women				Mothers-in-law				Household head			
	Ate any of the food group		Intake, g, if any consumed		Ate any of the food group		Intake, g, if any consumed		Ate any of the food group		Intake, g, if any consumed	
Food group^a^	*n*	(%)	Mean	(SD)	*n*	(%)	Mean	(SD)	*n*	(%)	Mean	(SD)
Starchy staples	150	(100)	896	(319)	150	(100)	886	(367)	149	99	1098	(427)
Pulses (beans, peas and lentils)	143	(95)	96	(57)	142	(95)	96	(56)	140	93	113	(69)
Nuts and seeds	47	(31)	6.3	(21)	52	(35)	6.7	(13)	53	35	6.6	(20)
Dairy	102	(68)	257	(224)	91	(61)	240	(197)	109	73	324	(272)
Flesh foods (meat, fish and shellfish)	49	(33)	52	(53)	48	(32)	57	(43)	65	43	73	(61)
Eggs	27	(18)	4.8	(12)	30	(20)	7.9	(26)	42	28	7.1	(20)
Green leafy vegetables	100	(67)	25	(31)	103	(69)	24	(29)	102	68	22	(24)
Other vitamin A-rich fruits and vegetables	103	(69)	201	(239)	105	(70)	226	(282)	96	64	214	(239)
Other vegetables	147	(98)	158	(103)	150	(100)	154	(95)	148	99	189	(122)
Other fruits	40	(27)	52	(47)	33	(22)	55	(65)	35	23	32	(28)
Alcohol	0	(0)	0	(0)	0	(0)	0	(0)	24	16	45	(111)
% (*n*) consuming ≥5 groups; mean dietary diversity^b^	87	(58)	4.6	(1.2)	88	(59)	4.7	(1.3)	93	62	4.9	(1.3)

^a^ Intakes based on average over 3-day recall period

^b^ Dietary diversity score based on 1-day recall period

Table 3 reports the tests for equality in log-FS and log-FS:ES. Women (pregnant women and mothers-in-law) had lower dietary diversity and intakes of starchy foods, pulses, vegetables and animal-source foods than male household heads. Comparing log-FS:ES, a larger share of women’s than men’s diets was provided by starchy foods, pulses, vitamin A-rich fruits and vegetables, and green leafy vegetables. Pregnant women had 34% higher shares of green leafy vegetables. Men’s diets comprised 18% larger shares of flesh foods than pregnant women and 24% larger shares of dairy than the mothers-in-law. Log-FS and log-FS:ES were not different between pregnant women and mothers-in-law (*P* > 0.4 for all foods; results not shown).

**Table 3:** Differences in food shares (FS) and food-shares-to-energy shares (FS:ES) for each food group, between different pairs of household members who ate any of each food group

	Log FS			Log FS:ES			
Food group^a^	*n*	Mean	95% CI	*P*	Mean	95% CI	*P*
*Pregnant woman : household head*							
Starchy staples	149	−0.21	(−0.28, −0.13)	<0.001	0.05	(−0.00, 0.10)	0.068
Pulses (beans, peas and lentils)	137	−0.11	(−0.22, −0.00)	0.047	0.14	(0.03, 0.26)	0.017
Nuts and seeds	35	−0.05	(−0.33, 0.22)	0.70	0.14	(−0.11, 0.39)	0.271
Dairy	88	−0.31	(−0.53, −0.10)	0.004	−0.07	(−0.30, 0.17)	0.578
Flesh foods (meat, fish and shellfish)	43	−0.44	(−0.62, −0.26)	<0.001	−0.20	(−0.37, −0.04)	0.015
Eggs	24	−0.47	(−0.81, −0.12)	0.007	−0.28	(−0.63, 0.07)	0.115
Green leafy vegetables	94	0.00	(−0.16, 0.25)	0.69	0.29	(0.06, 0.52)	0.012
Other vitamin A-rich fruits and vegetables	79	0.23	(−0.13, 0.59)	0.20	0.47	(0.10, 0.83)	0.012
Other vegetables	146	−0.22	(−0.31, −0.14)	<0.001	0.00	(−0.06, 0.11)	0.593
Other fruits	21	0.53	(−0.68, 1.73)	0.39	0.74	(−0.42, 1.90)	0.213
Dietary diversity^b^	149	−0.07	(−0.11, −0.03)	0.001	−0.06	(−0.11, −0.01)	0.022
*Mother-in-law : household head*							
Starchy staples	149	−0.23	(−0.33, −0.13)	<0.001	0.00	(−0.00, 0.08)	0.07
Pulses (beans, peas and lentils)	136	−0.15	(−0.30, −0.01)	0.035	0.12	(0.02, 0.22)	0.017
Nuts and seeds	39	−0.28	(−0.62, 0.06)	0.107	0.00	(−0.36, 0.30)	0.84
Dairy	81	−0.47	(−0.67, −0.27)	<0.001	−0.28	(−0.47, −0.08)	0.005
Flesh foods (meat, fish and shellfish)	40	−0.38	(−0.65, −0.10)	0.008	−0.13	(−0.37, 0.10)	0.27
Eggs	27	−0.34	(−0.63, −0.06)	0.019	−0.12	(−0.42, 0.19)	0.45
Green leafy vegetables	95	0.00	(−0.20, 0.24)	0.862	0.29	(0.07, 0.52)	0.011
Other vitamin A-rich fruits and vegetables	83	0.32	(−0.18, 0.82)	0.213	0.55	(0.09, 1.02)	0.020
Other vegetables	148	−0.24	(−0.33, −0.14)	<0.001	0.00	(−0.06, 0.12)	0.56
Other fruits	17	0.32	(−0.96, 1.60)	0.623	0.57	(−0.77, 1.91)	0.41
Dietary diversity^b^	149	−0.07	(−0.13, −0.01)	0.013	−0.02	(−0.09, 0.05)	0.54

^a^ Intakes based on average over 3-day recall period; kcal intakes adjusted for by calculating food share to energy share [FS:ES between persons a and b=(intake_a_/kcal_a_)/(intake_b_/kcal_b_)]

^b^ Dietary diversity score is based on 1-day recall period, and ‘log-FS’ was the log-transformed ratio between dietary diversity scores, whereas ‘log-FS:ES’ used the same log-dietary diversity ratio but adjusted for the corresponding log-transformed kcal intake ratios.

Intakes, EARs and PAs for each household member are reported in Table 4. Pregnant women had the lowest MPA (37%) compared with mothers-in-law (52%) and male household heads (57%). Vitamin B_12_ intakes were inadequate for almost all respondents.

**Table 4:** Daily estimated average requirements, nutrient intakes and probability of adequacy by household member

	Pregnant women							
	Requirements^a^		Intakes^b^			Probability of adequacy, %		
Nutrient	EAR	(SD)	Mean	(SD)	Median	Mean	(SD)	Median
*Pregnant women*								
Energy, kJ/d	–	–	9372	(3056)	8983	–	–	–
Energy, kcal/d	–	–	2239	(730)	2146	–	–	–
Protein, g/d	–	–	68	(24)	65	–	–	–
Vitamin C, mg/d	40	(4.0)	133	(144)	96	91	(24)	100
Vitamin A, RE	370	(74)	486	(449)	359	17	(25)	7
Thiamin, mg/d	1.2	(0.1)	1.5	(0.7)	1.5	65	(39)	86
Riboflavin, mg/d	1.2	(0.1)	1.1	(0.6)	1.0	20	(34)	0
Niacin, mg/d	14	(2.1)	16	(7.1)	15	54	(36)	53
Vitamin B_6_, mg/d	1.6	(0.2)	2.2	(0.8)	2.1	79	(33)	99
Folate, µg/d	520	(52)	639	(624)	325	24	(40)	0
Vitamin B_12_, µg/d	2.2	(0.2)	0.8	(0.9)	0.4	0	(0.0)	0
Iron, mg/d^c^	22	(2.1)	25	(25)	17	20	(36)	0
Zinc, mg/d^d^	12	(1.5)	11	(4.0)	11	29	(33)	10
Calcium, mg/d^e^	800	(100)	654	(462)	505	14	(31)	0
Mean PA	–	–	–	–	–	37	(20)	36
*Mothers-in-law*								
Energy, kJ/d	–	–	9326	(3324)	9163	–	–	–
Energy, kcal/d	–	–	2228	(794)	2189	–	–	–
Protein, g/d	–	–	67	(28)	65	–	–	–
Vitamin C, mg/d	30	(3.0)	138	(136)	98	96	(17)	100
Vitamin A, RE	270	(54)	511	(646)	333	40	(38)	29
Thiamin, mg/d	0.9	(0.1)	1.5	(0.7)	1.4	88	(28)	100
Riboflavin, mg/d	0.9	(0.1)	1.0	(0.6)	0.9	39	(41)	17
Niacin, mg/d	11	(1.7)	16	(7.2)	16	79	(32)	99
Vitamin B_6_, mg/d	1.1	(0.1)	2.2	(0.8)	2.1	100	(0)	100
Folate, µg/d	320	(32)	350	(165)	325	34	(38)	14
Vitamin B_12_, µg/d	2	(0.2)	0.6	(2.1)	0.3	0	(0)	0
Iron, mg/d^c^	–	–	15	(7)	14	2.7	(7.6)	0
Zinc, mg/d^d^	7	(0.9)	11	(5)	11	87	(28)	100
Calcium, mg/d^e^	800	(100)	511	(277)	434	6.2	(20)	0
Mean PA	–	–	–	–	–	52	(16)	51
*Male household heads*								
Energy, kJ/d	–	–	11,892	(3692)	12,085	–	–	–
Energy, kcal/d	–	–	2841	(882)	2887	–	–	–
Protein, g/d	–	–	87	(29)	84	–	–	–
Vitamin C, mg/d	40	(4.0)	128	(105)	91	90	(27)	100
Vitamin A, RE	300	(60)	502	(402)	355	45	(38)	36
Thiamin, mg/d	1	(0.1)	2.0	(1.0)	1.9	95	(19)	100
Riboflavin, mg/d	1	(0.1)	1.3	(0.7)	1.2	65	(40)	88
Niacin, mg/d	12	(1.8)	22	(9.8)	21	95	(17)	100
Vitamin B_6_, mg/d	1.1	(0.1)	2.8	(1.0)	2.7	99	(9)	100
Folate, µg/d	320	(32)	402	(158)	385	60	(41)	77
Vitamin B_12_, µg/d	2	(0.2)	0.9	(1.2)	0.6	2.7	(15)	0.0
Iron, mg/d^c^	–	–	19	(6.7)	19	25	(24)	20
Zinc, mg/d^d^	15	(1.9)	14	(4.7)	14	29	(31)	16
Calcium, mg/d^e^	800	(100)	686	(407)	597	16	(32)	0.1
Mean PA	–	–	–	–	–	57	(17)	60

^a^ EARs using WHO/FAO values (33), unless otherwise stated

^b^ Intakes reported as mean intakes, averaged across the three dietary recalls

^c^ Institute of Medicine values for iron (35). We assumed low bioavailability of iron (5%), except for iron in pregnant women who have higher absorption (23%) during pregnancy. Iron probabilities of adequacy for mothers-in-law and men were calculated using a table of probabilities for different intervals of usual intakes, adapted from IOM but assuming 5% bioavailability

^d^ Based on International Zinc Nutrition Consultative Group (IZiNCG) recommendations (36). We assumed a low bioavailability of zinc (25% absorption for women; 18% for men)

^e^ Institute of Medicine values for calcium (34).

### Testing for equity and the determinants of equity

Table 5 reports calorie (log-transformed log-RDEARs) and micronutrient (log-transformed MPA ratios) allocations, and determinants of these outcomes. We focus on allocation between pregnant women and other household members because of the nutritional importance of diet during pregnancy.

**Table 5:** Tests for intra-household equity and the determinants of inequity in the allocation of energy (RDEARs) and nutrients (MPA ratios) using multivariable linear regression

	Pregnant woman : household head			Pregnant woman : mother-in-law		
	Coeff.	(95% CI)	*P*	Coeff.	(95% CI)	*P*
*log-RDEAR*						
Crude mean outcome (*n* = 149)	−0.20	(−0.26, 0.15)	<0.001	−0.15	(−0.22, −0.07)	<0.001
*n* (fitted in multivariable model)	145			145		
Earning disparities between pregnant women and their spouse						
Earns less than the spouse	Ref			Ref		
Earns more or same as the spouse	0.27	(0.12, 0.42)	<0.001	0.16	(0.02, 0.30)	0.023
Number of previous pregnancies						
0	Ref			Ref		
≥1	−0.01	(−0.13, 0.11)	0.88	0.04	(−0.08, 0.15)	0.52
Empowerment						
Self-reported empowerment level	0	(−0.02, 0.03)	0.78	0.02	(−0.01, 0.04)	0.16
Food security						
Asset score	0.03	(−0.01, 0.06)	0.15	−0.01	(−0.04, 0.03)	0.75
Household mean intakes per capita	0.13	(0.04, 0.22)	0.007	−0.02	(−0.10, 0.07)	0.70
Husband working overseas						
Not working overseas	Ref			Ref		
Working overseas	−0.06	(−0.20, 0.08)	0.39	0.14	(0.01, 0.27)	0.035
Caste/religious group						
Dalit or Muslim (disadvantaged)	Ref			Ref		
Janajati/other Terai castes	0.05	(−0.08, 0.17)	0.49	0.08	(−0.04, 0.20)	0.19
Yadav/Brahmin (least disadvantaged)	−0.04	(−0.20, 0.12)	0.60	−0.04	(−0.19, 0.10)	0.56
Season						
Pre-monsoon	Ref			Ref		
Monsoon	−0.03	(−0.14, 0.08)	0.58	−0.02	(−0.12, 0.08)	0.71
*log-MPA ratio*						
Crude mean outcome (*n* = 149)	−0.47	(−0.72, −0.22)	<0.001	−0.54	(−0.76, −0.31)	<0.001
*n* (fitted in multivariable model)	144			145		
Earning disparities between pregnant women and their spouse						
Earns less than the spouse	Ref			Ref		
Earns more or same as the spouse	−0.05	(−0.80, 0.70)	0.90	0.14	(−0.18, 0.46)	0.39
Number of previous pregnancies						
0	Ref			Ref		
≥1	0.29	(−0.32, 0.90)	0.35	0.08	(−0.19, 0.34)	0.56
Empowerment						
Self-reported empowerment level	−0.03	(−0.15, 0.08)	0.57	0.00	(−0.05, 0.05)	0.89
Food security						
Asset score	0.03	(−0.14, 0.21)	0.71	0.02	(−0.05, 0.10)	0.55
Household mean intakes per capita	0.07	(−0.39, 0.52)	0.78	0.43	(0.23, 0.63)	<0.001
Husband working overseas						
Not working overseas	Ref			Ref		
Working overseas	0	(−0.69, 0.68)	0.99	0.22	(−0.07, 0.52)	0.14
Caste/religious group						
Dalit or Muslim (disadvantaged)	Ref			Ref		
Janajati/other Terai castes	0.33	(−0.31, 0.97)	0.32	0.2	(−0.08, 0.48)	0.16
Yadav/Brahmin (least disadvantaged)	0.89	(0.11, 1.67)	0.026	0.25	(−0.09, 0.59)	0.14
Season						
Pre-monsoon	Ref			Ref		
Monsoon	0.18	(−0.37, 0.72)	0.53	−0.06	(−0.30, 0.17)	0.60

Variance inflation factors ≤ 1.5

Between pregnant women and household heads, RDEARs were 18% lower, and MPA ratios 38% lower, than perfectly equitable households. Between pregnant women and mothers-in-law, RDEARs were 14% lower, and MPA 42% lower, than perfect equity. In 17% of households, pregnant women consumed <90% of EARs, while the household heads consumed >110% of EARs. In 11% of households, pregnant women consumed <90% of EARs, while mothers-in-law consumed >110% of EARs.

RDEARs were positively associated with women earning the same or more than their spouse, and the pregnant woman’s husband living overseas. Household-level intakes were associated with MPA ratios. There was no evidence of a nonlinear relationship between wealth and calorie or micronutrient allocation, as there was no association with a quadratic term or when testing different quintiles.

## Discussion

Foods and nutrients are allocated inequitably within households, with clear male advantage. Male household heads consume more animal-source foods, eat special foods like deep-fried snacks and alcohol outside of the home, and have the highest dietary adequacy, whereas women eat more low-status foods and have lower dietary adequacy, particularly pregnant women due to their elevated requirements. The intra-household gradient in dietary adequacy (men>mothers-in-law>pregnant women) mirrors the gradient in MUAC and is determined by within-household disparities in earned cash income, pregnant woman’s husband working overseas, and household-level calorie consumption.

The gender division in food allocation is consistent with other studies from Nepal. One study found that men were preferentially allocated ‘luxury’ foods such as tea and deep-fried snacks [43], and another found that men had higher micronutrient adequacy than women [11]. We found no clear disparity in food allocation between pregnant women and their mothers-in-law, which is surprising given the well-reported social hierarchy between women in South Asia [44]. However, pregnant women’s intakes were less adequate because their elevated requirements were not compensated for, perhaps due to male favouritism, fear of giving birth to a large baby, fasting for a boy child [13], food proscriptions [7] or feeling full since women were in their third trimester [45].

We found higher nutrient intakes than studies from urban Nepal [2] and rural Bangladesh [1]. This may be because rural populations eat more, because they engage in physically strenuous agricultural labour, whereas urban populations may be more sedentary. We did not measure the physical activity levels of respondents, beyond a basic self-assessment of activity levels, nor did these other studies, so, we cannot determine whether differences in workloads could explain these differences in dietary intakes. Future work could examine urban–rural differences, and improve the accuracy of these dietary adequacy estimates (particularly calorie adequacy ratios and RDEARs), by incorporating the use of accelerometers to quantify energy balance. During data collection, we also noticed some very high intakes, which interviewers explained were due to Muslims feasting after sunset. Only 13% of our sample was Muslim, and analyses without fasting and feasting households gave similar results.

Other variance between studies may be explained by temporal and methodological differences, such as different dietary assessment methods. We used a repeated 24-h recall method using a photographic atlas to estimate portion sizes, whereas other studies from Nepal and Bangladesh used weighed food records over a 24-h recall period [2], and/or direct observations [1, 11] to measure diets. Ideally, we would have used weighed methods to give a continuous measure of portion sizes (rather than the categorical measure introduced by the atlas), and also used observations rather than recall-based methods to reduce error introduced by respondents’ inaccuracies in their conceptualisation and recall of portion sizes [46, 47]. During pilot testing, we found that direct observations were not feasible because they were time consuming and burdensome on respondents. Also, it was culturally inappropriate for male interviewers to spend long periods of time in or near the kitchen with the female cook, making both weighed and observational methods difficult for male interviewers. The few female interviewers we did employ (few local women were sufficiently qualified) were not permitted to spend nights away from home or travel in the dark to conduct direct observations [24]. Nevertheless, our validation study, which found moderate agreement between portion sizes that were weighed and estimated 24-h later using a photographic atlas [24], gives us some confidence in our dietary intake estimates.

Relative cash incomes predicted intra-household calorie allocations, which is consistent with the limited evidence on this association [16], and could be due to perceptions of deservedness [12], a way of rewarding earners [48] or because nutritional investment in economically productive members yields higher incomes [49]. We found an association between household-level calorie consumption and micronutrient allocation but not calorie allocation; other studies have also found no association between food security and calorie allocation [16]. The association between husbands living overseas and food allocation may be explained by women receiving overseas remittances, although a study from the same district found that women worried about the care they would receive from in-laws when their husbands were away [50].

The external validity is limited by our selective sampling of joint, male-headed households, sampling only three respondents within each household, and the 4-month survey period, although we found no effect of season on food allocation. We focused on comparisons between pregnant women and household members who we hypothesised to be favoured in the allocation of foods, and who we hoped would change their behaviours due to our intervention. However, this prevented us from comparing pregnant women with less senior household members (such as children, adolescents or more junior non-pregnant women), who might also be nutritionally vulnerable.

There are a few limitations in the analyses. We are unable to attribute causality to the associations, and are also limited by the sample size. Using data from all study arms could have increased statistical power, but we anticipated interactions between the predictors and study arm. To limit non-response, pregnant woman sometimes answered on behalf of others (34% and 37% of household heads, and 17% and 21% of mothers-in-law, in the second and third visits, respectively). Therefore, food eaten outside may have been missed. If so, dietary intakes of mothers-in-law and male household heads, as well as allocation ratios, would be underestimated. This was particularly concerning for the 73% of household heads who consumed at least some food outside of the home. However, we found no significant differences between self-reported and proxy-estimated calorie intakes, suggesting that any bias introduced by using a proxy respondent is likely to be minimal. Using standard rather than individual recipes might have falsely reduced variance in intakes, but are unlikely to have affected allocation estimates; whereas, it is possible that not applying retention factors biased the adequacy ratios, if certain household members consumed systematically more raw or cooked foods.

Our findings can be used to predict how interventions might influence intra-household food allocation. General increases in food security could increase nutritional equity, but programmes increasing availability of low-status foods (such as green leafy vegetables) could disproportionately benefit women, while increasing availability of animal-source foods may disproportionately benefit men. This hypothesis is supported by two Bangladeshi studies. One found that vegetables promoted in a gardening intervention, that were considered inferior, were selectively channelled to women [51]; another found that rice transfers (high status) were disproportionately consumed by men, whereas wheat transfers (low status) were channelled to women [52]. Furthermore, numerous kitchen garden interventions have improved women’s consumption of fruits and vegetables [15], whereas livestock programmes have produced mixed effects on consumption of animal-source foods [15, 53]. Programmes targeting women could try to influence perceptions about the status of foods, and (preferably) also influence women’s sociocultural status, although qualitative research is needed to understand how these changes in perceptions could be achieved. Beyond these gender dynamics, we can also predict how interventions might affect allocation to pregnant women specifically. Given that household-level calorie consumption was positively associated with higher equity for pregnant women, these above-mentioned interventions may selectively benefit pregnant women simply by increasing household-level food availability. Other interventions to increase pregnant women’s relative cash income, such as employment opportunities, higher wages or cash transfers, might also increase the allocation of foods to pregnant women. If so, a crucial next step would be to explore how these income-generating interventions can benefit women without adding to their work burdens, energy expenditure or compromising their ability to care for themselves and their children.
